# Supplementary material for: Cortical overgrowth in a preclinical forebrain organoid model of CNTNAP2-associated autism spectrum disorder
Source: Nat Commun. 2021 Sep 1;12:4087. doi: 10.1038/s41467-021-24358-4 (PMC8410758; doi:10.1038/s41467-021-24358-4)
Supplement: Supplementary file 1 — Supplementary Information [file 41467_2021_24358_MOESM1_ESM.pdf]

Supplementary figure 1.

A

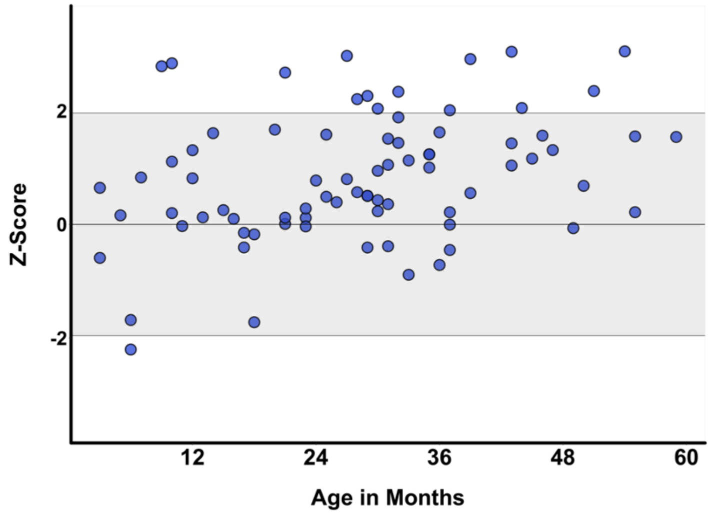

B

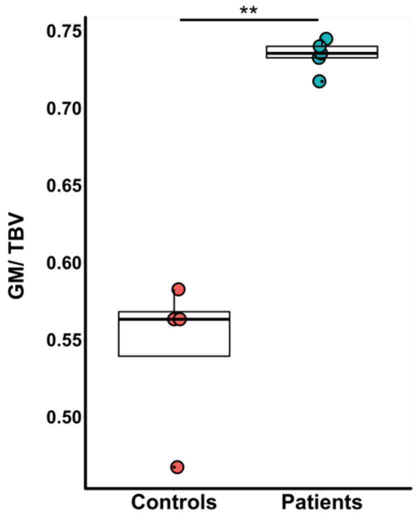

C

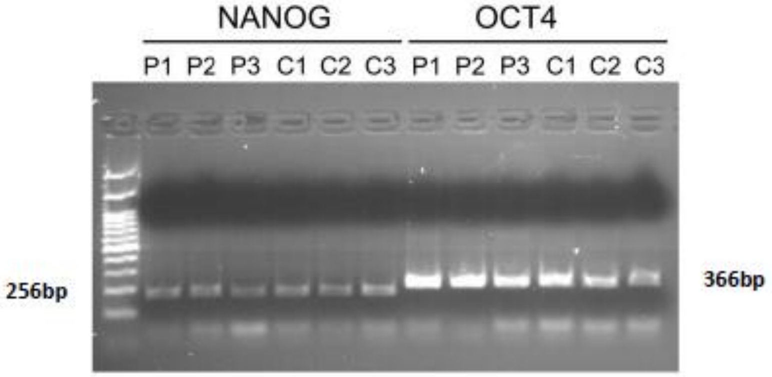
NANOG
OCT4
P1 P2 P3 C1 C2 C3 P1 P2 P3 C1 C2 C3
256bp
366bp

D

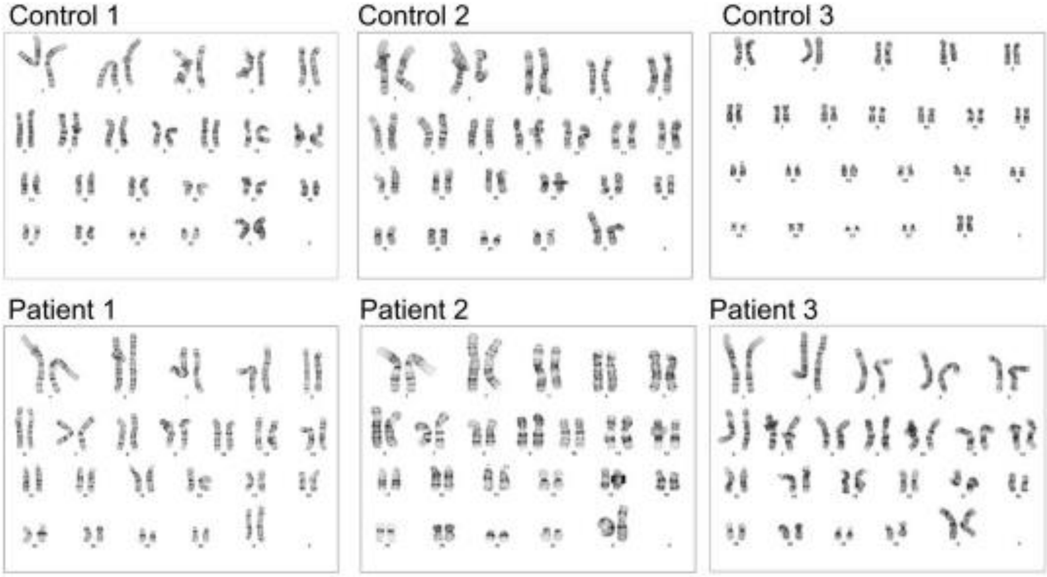
Control 1
Control 2
Control 3
Patient 1
Patient 2
Patient 3

E

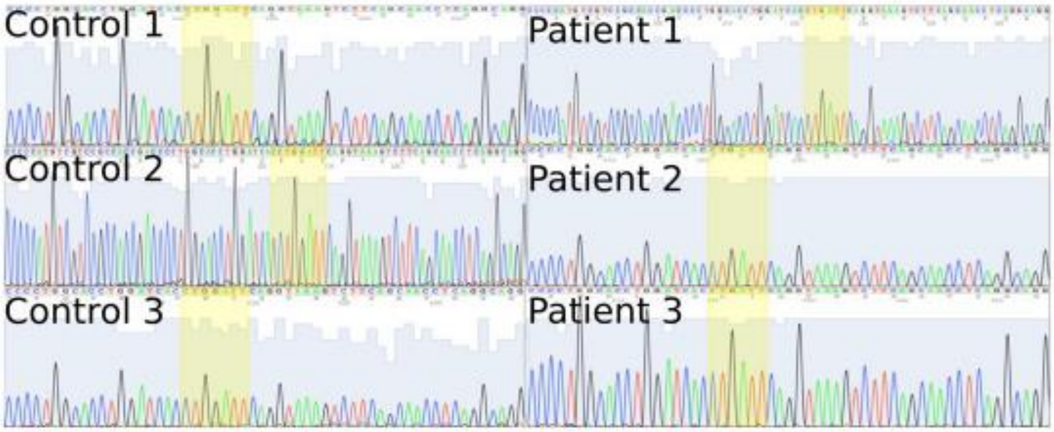
Control 1
Patient 1
Control 2
Patient 2
Control 3
Patient 3

F

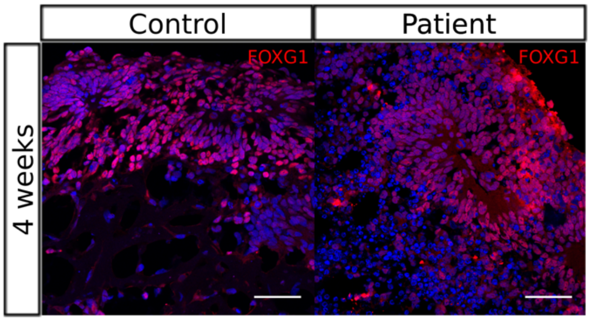
Control
Patient
4 weeks
FOXG1
FOXG1

G

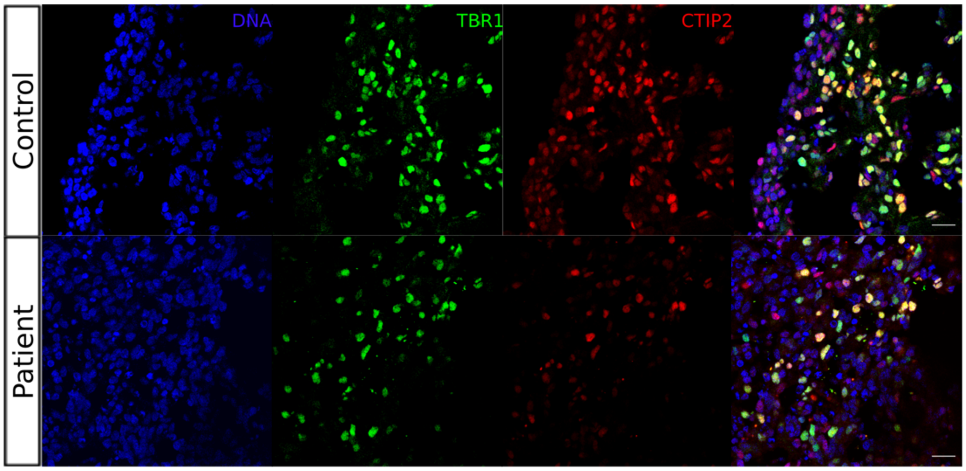
DNA
TBR1
CTIP2
Control
Patient

H

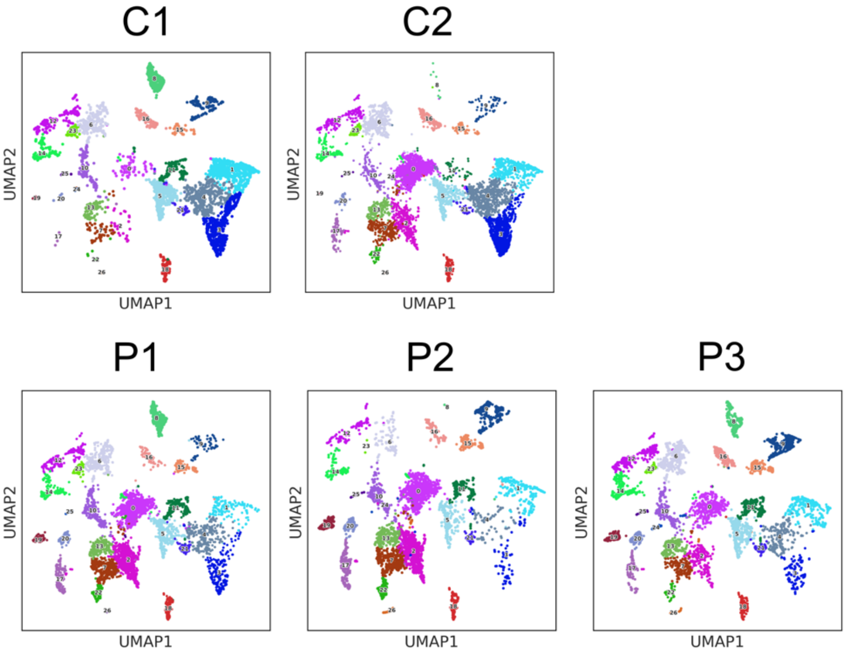
C1
C2
P1
P2
P3
UMAP1
UMAP2

I

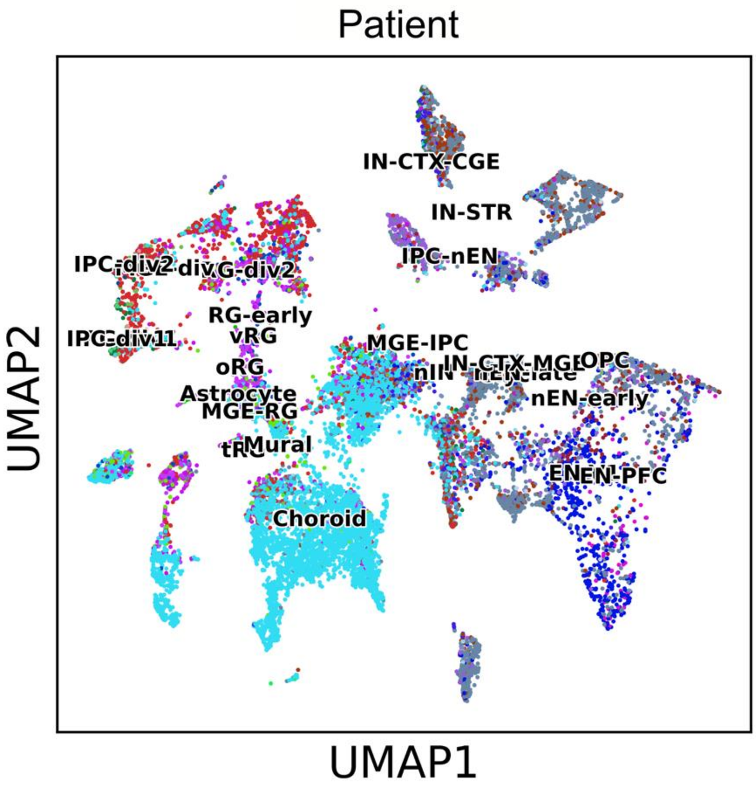
Patient
IN-CTX-CGE
IN-STR
IPC-nEN
RG-early
vRG
oRG
Astrocyte
MGE-RG
Mural
MGE-IPC
nIN
nEN-early
OPC
Choroid
UMAP2
UMAP1

J

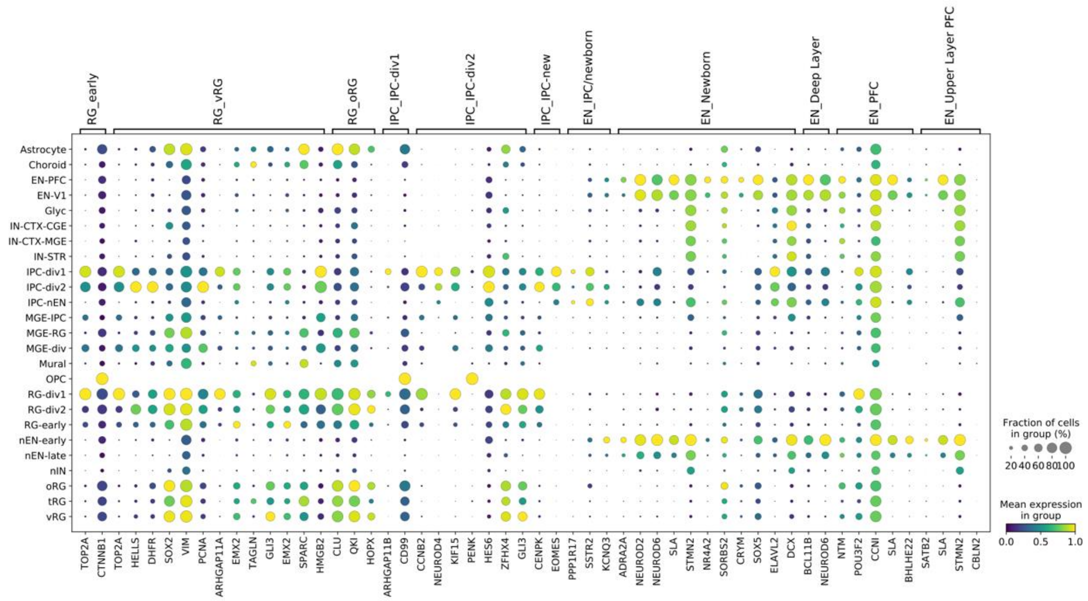
RG_early
RG_vRG
RG_oRG
IPC_IPC-div1
IPC_IPC-div2
IPC_IPC-new
EN_IPC/newborn
EN_Newborn
EN_Deep Layer
EN_PFC
EN_Upper Layer PFC
Astrocyte
Choroid
EN-PFC
EN-V1
Glyc
IN-CTX-CGE
IN-CTX-MGE
IN-STR
IPC-div1
IPC-div2
IPC-nEN
MGE-IPC
MGE-RG
MGE-div
Mural
OPC
RG-div1
RG-div2
RG-early
nEN-early
nEN-late
nIN
oRG
tRG
vRG
TOP2A CTNNB1 TOP2A HELLS DHFR SOX2 VIM PCNA ARHGAP11A EMX2 TAGLN GLI3 EMX2 SPARC HMGB2 CLU QKI HOPX ARHGAP11B CD99 CCNB2 NEUROD4 KIF15 PENK HES6 ZFHX4 GLI3 CENPK EOMES PPP1R17 SSTR2 KCNQ3 ADRA2A NEUROD2 NEUROD6 SLA STMN2 NR4A2 SORBS2 CRYM SOX5 ELAVL2 DCX BCL11B NEUROD6 NTM POU3F2 CCNI SLA BHLHE22 SATB2 SLA STMN2 CBLN2
Fraction of cells in group (%)
20 40 60 80 100
Mean expression in group
0.0 0.5 1.0

K

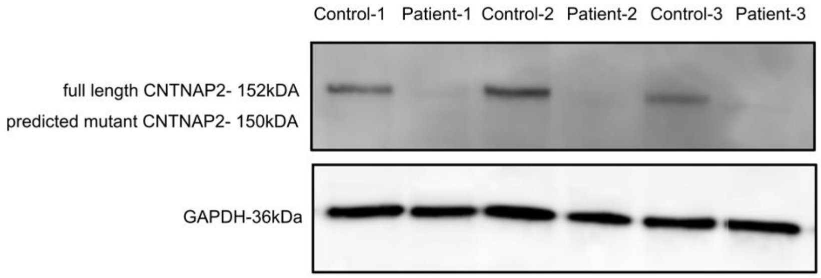

Control-1
Patient-1
Control-2
Patient-2
Control-3
Patient-3
full length CNTNAP2- 152kDA
predicted mutant CNTNAP2- 150kDA
GAPDH-36kDa

L

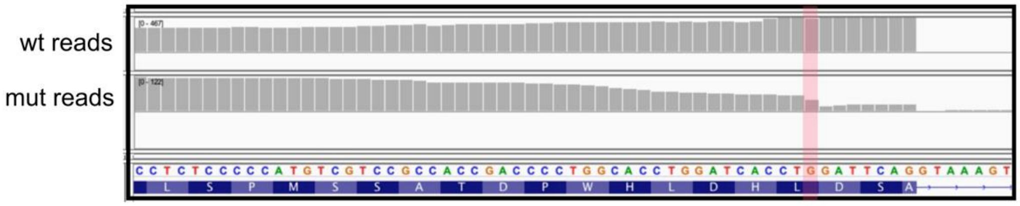

wt reads
mut reads
CCTCTCCCCCATGTCGTCCGCCACCGACCCCTGGCACCTGGATCACCTGGATTCAGGTAAAGT
L S P M S S A T D P W H L D H L D S A

M

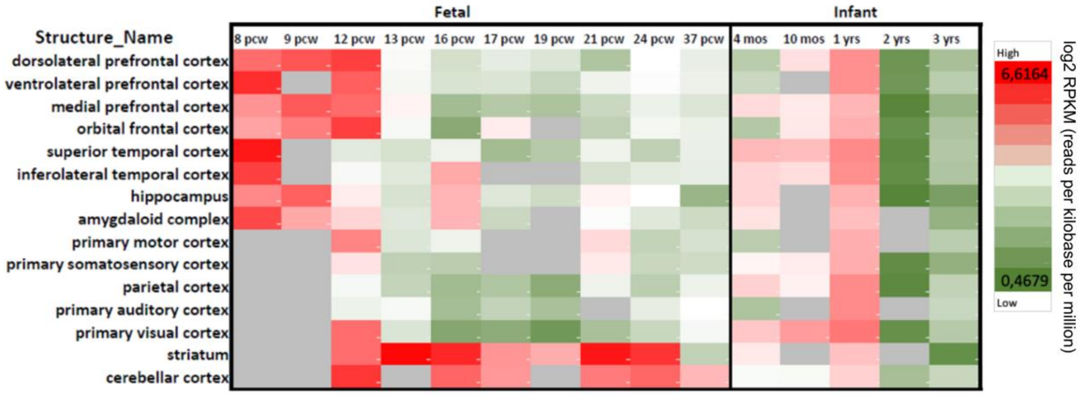

Fetal
Infant
Structure_Name
8 pcw
9 pcw
12 pcw
13 pcw
16 pcw
17 pcw
19 pcw
21 pcw
24 pcw
37 pcw
4 mos
10 mos
1 yrs
2 yrs
3 yrs
dorsolateral prefrontal cortex
ventrolateral prefrontal cortex
medial prefrontal cortex
orbital frontal cortex
superior temporal cortex
inferolateral temporal cortex
hippocampus
amygdaloid complex
primary motor cortex
primary somatosensory cortex
parietal cortex
primary auditory cortex
primary visual cortex
striatum
cerebellar cortex
High
6,6164
0,4679
Low
log2 RPKM (reads per kilobase per million)

N

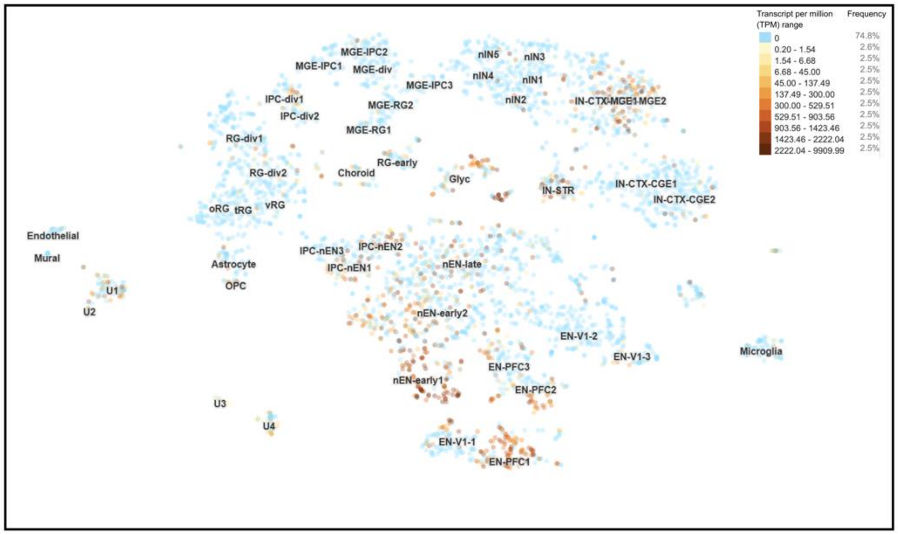

Transcript per million (TPM) range
Frequency
0
0.20 - 1.54
1.54 - 6.68
6.68 - 45.00
45.00 - 137.49
137.49 - 300.00
300.00 - 529.51
529.51 - 903.56
903.56 - 1423.46
1423.46 - 2222.04
2222.04 - 9909.99
MGE-IPC2
MGE-IPC1
MGE-div
MGE-IPC3
MGE-RG2
MGE-RG1
IPC-div1
IPC-div2
RG-div1
RG-div2
Choroid
RG-early
nIN5
nIN3
nIN4
nIN1
nIN2
IN-CTX-MGE1
MGE2
Glyc
IN-STR
IN-CTX-CGE1
IN-CTX-CGE2
oRG
tRG
vRG
Endothelial
Mural
U1
U2
Astrocyte
OPC
IPC-nEN3
IPC-nEN2
IPC-nEN1
nEN-late
nEN-early2
EN-V1-2
EN-V1-3
Microglia
EN-PFC3
EN-PFC2
nEN-early1
U3
U4
EN-V1-1
EN-PFC1

Supplementary figure 1 legend.

A. Longitudinal head circumference measurements were obtained from 20 unique female and 17 unique male pediatric patients who were between birth and 5 years old, all from the Old-Order Amish community, diagnosed with ASD and carrying the c.3709DelG mutation in *CNTNAP2.* Z-scores were calculated using average head circumference measurements per month and gender and mean and SD from WHO-reference data (1) (weighted z-score = 2.93, p <0.003).

B. Quantification of total gray matter (GM) relative to total brain volume (TBV) of 6 patients carrying the homozygous c.3709DelG mutation in *CNTNAP2* compared to 4 age-matched control templates, each of which is based on a composite of MRIs from healthy individuals for that given age. (Patients: GM/TBV= 0.73; Controls: GM/TBV= 0.54;two-sided t-test, p = 0.004, n= 6 patients, 4 control templates) Boxplots display median, first and third quartiles, and whiskers showing the largest and smallest values no further than 1.5 times the inter quartile range from first and third quartile, respectively. * = p < 0.05; ** = p < 0.01; *** = p < 0.001.

C. qRT-PCR shows the presence of stemness markers NANOG and OCT4 in all iPSC lines. This experiment was not repeated independently.

D. Karyotyping analysis of all patient- and control-derived hiPSC lines indicates that the iPSCs derived from each subject have normal karyotype.

E. Sanger sequencing analysis of the genomic fragment surrounding the mutation site confirms all iPSC lines have the correct *CNTNAP2* genotypes. All three patient-derived iPSCs show a single guanine deletion at cDNA position 3709, the three control lines show 2 guanines at the same position. Three surrounding base pairs upstream and downstream of mutation site marked in yellow.

F. Examples of immunohistochemistry images of 4-week-old cortical organoid sections showing expression of dorsal forebrain specific transcription factor *FOXG1* in both control- and patient-derived organoids. All control- and patient-derived organoids displayed similar expression patterns for this marker. Scale bar represents 50 µm.

G. Representative examples of immunohistochemistry images of 13 week-old cortical organoid sections showing expression of early born neuron layer markers TBR1 and CTIP2. All control- and patient-derived organoids displayed similar expression patterns for these markers. Scale bar represents 20 µm.

H. UMAP plots displaying the cell clusters determined by the unsupervised Leiden clustering method (see methods for details) for all individual control- and patient-derived organoids, showing that all 26 clusters are consistently present in all individual samples.

I. UMAP plot of cell lineage distribution annotated according to Nowakowski et al. (2017)(2) in patient-derived brain organoids (n=3 samples, 16,780 cells).

J. Dot plot displaying expression levels of signature markers expressed annotated cell types, present in the all forebrain organoids at 13 weeks *in vitro*. Color of dots represents mean gene expression level; size of dots represents fraction of cells in cell type expressing the gene.

K. Western blot analysis showing presence of full length CNTNAP2 protein levels in all control-derived organoids and absence in all patient-derived cortical organoids using an antibody directed against the last Laminin G domain positioned before the transmembrane domain (Antibody B in Fig. 1A), indicating there is no truncated protein (predicted size ~150kDa) present in patient-derived organoids. This experiment was repeated independently and generated similar results.

L. Aligned mRNA sequence reads from scRNAseq of control- and patient derived organoids, showing lower read coverage for the patient-derived organoid at the 3'-end of the mRNA, suggesting that a nonsense-mediated decay occurs in *CNTNAP2* mRNA in patient organoids.

M. Heat map displaying *CNTNAP2* expression levels from human embryonic RNA sequencing data that are publicly available at www.brainspan.com (3). Expression is highest in multiple cortical regions, amygdala and hippocampus in the first 12 weeks post conception, and in striatum and cerebellum between 12- and 37 weeks post conception.

N. t-SNE plot obtained from https://cells.ucsc.edu/?ds=cortex-dev showing the *CNTNAP2* mRNA levels in all cell lineages in developing human telencephalon, as annotated by Nowakowski *et al* (2017) (2). *CNTNAP2* is highly expressed in different types of excitatory and inhibitory neurons, which is in line with the observation in our brain organoids.

Supplementary figure 2.

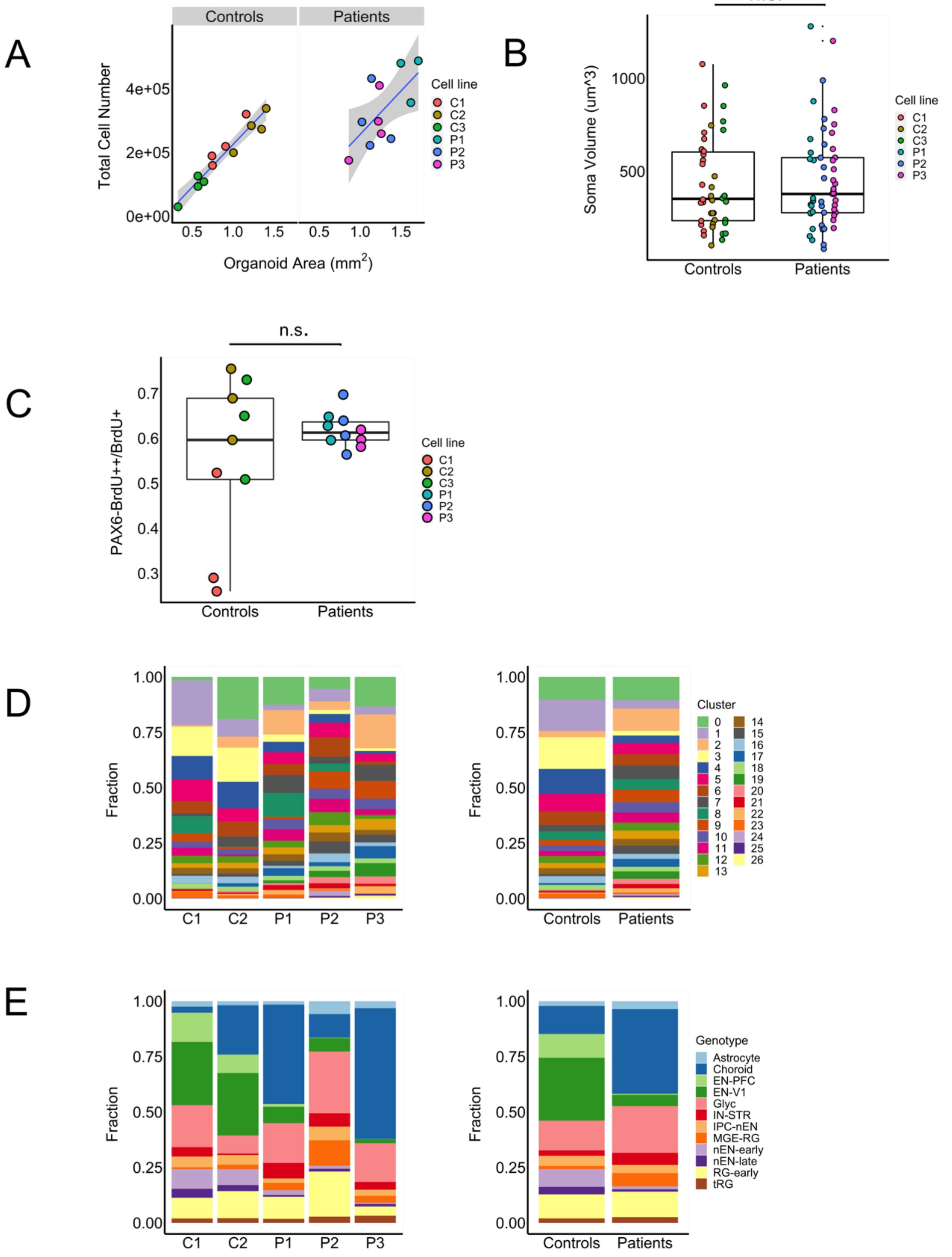

Supplementary figure 2 legend.

A. Plots of linear relationships between organoid projected surface area and total organoid cell number for both control- and patient-derived organoids at 13 weeks *in vitro*. Shaded grey area is 95% confidence interval estimate around the mean. (Linear model, Controls: $F(1,10)= 10.64$, $R^2= 0.52$, $p = 0.0085$. Patients: $F(1,9)=2.31$, $R^2= 0.34$, $p =0.16$, n= 3 cell lines, C1-C2-C3-P2-P3: n=4 orgs/cell line P1:n=3 orgs/cell_line). The shaded grey area represents the constructed 95% confidence interval around the mean.

B. Quantification of neuronal soma from organoids infected with AAV-Synapsin-GFP at 40 weeks showing no difference in neuronal somatic volume between control- and patient-derived cortical organoids (LRT, $\chi^2(1)= 0.16$, $p = 0.69$, n= 3 cell lines, 3 orgs/ cell line, 8 neuronal soma/org*)*. Boxplots display median, first and third quartiles, and whiskers showing the largest and smallest values no further than 1.5 times the inter quartile range from first and third quartile, respectively. * = $p < 0.05$; ** = $p < 0.01$; *** = $p < 0.001$.

C. Quantification of BrdU + cells that stained positive for PAX6 as well at 6 weeks *in vitro*, showing no difference between genotype groups (LRT, $\chi^2(1)= 0.54$, $p = 0.46$). Boxplots display median, first and third quartiles, and whiskers showing the largest and smallest values no further than 1.5 times the inter quartile range from first and third quartile, respectively. * = $p < 0.05$; ** = $p < 0.01$; *** = $p < 0.001$.

D. Stacked bar plot displaying relative cell fractions in each unsupervised Leiden cluster in patient (P1-3) and control (C1-C2) organoids as determined using scRNAseq for individual cell lines (left panel) and pooled per genotype (right panel).

E. Stacked bar plot displaying relative cell type fractions in patient (P1-3) and control (C1-C2) organoids as determined using scRNAseq and annotated using the cell types identified by Nowakowski et al. (2)for individual cell lines (left panel) and pooled per genotype (right panel).

Supplementary figure 3

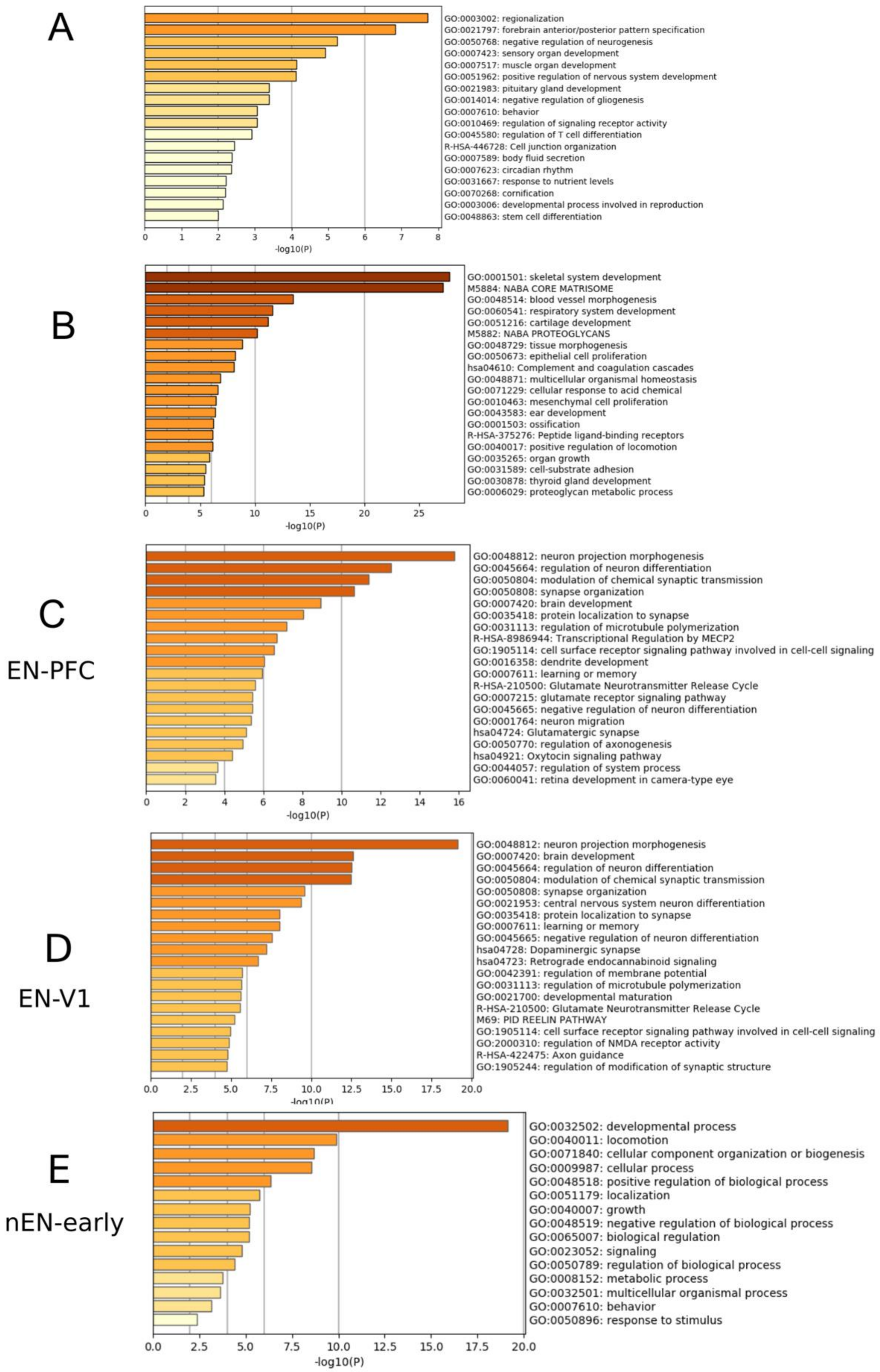

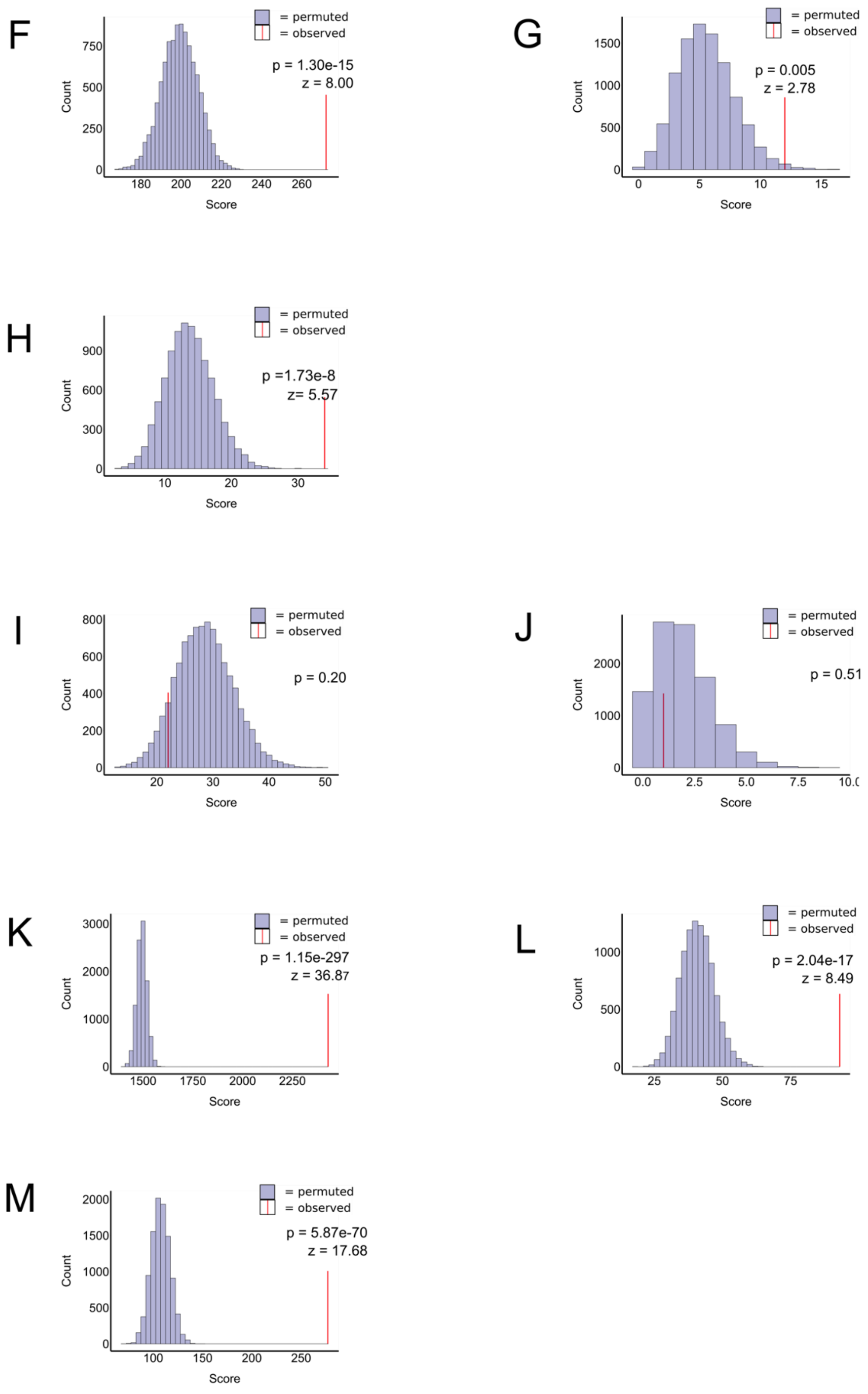
F
= permuted
= observed
p = 1.30e-15
z = 8.00
Count
Score
G
= permuted
= observed
p = 0.005
z = 2.78
Count
Score
H
= permuted
= observed
p =1.73e-8
z= 5.57
Count
Score
I
= permuted
= observed
p = 0.20
Count
Score
J
= permuted
= observed
p = 0.51
Count
Score
K
= permuted
= observed
p = 1.15e-297
z = 36.87
Count
Score
L
= permuted
= observed
p = 2.04e-17
z = 8.49
Count
Score
M
= permuted
= observed
p = 5.87e-70
z = 17.68
Count
Score

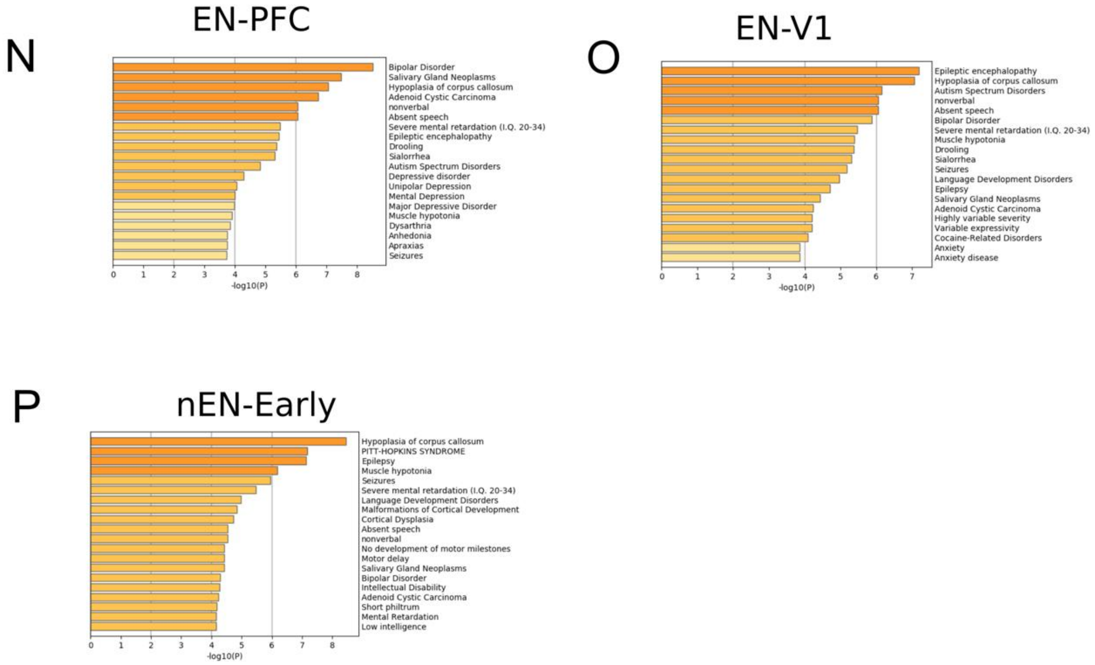

Supplementary figure 3 legend.

A and B. Gene Ontology (GO) analysis of differentially expressed (DE) genes in patient organoids determined by bulk RNA sequencing. The results show the biological process ontologies sorted by the smallest FDR p-value. Panel A shows GO analysis of differentially upregulated genes, panel B shows GO analysis downregulated genes.

C - E. Gene Ontology (GO) analysis of the top-100 most highly expressed genes in cell types with high CNTNAP2 expression in the control organoids: EN-PFC (C), EN-V1 (D), nEN-early (E) as annotated by single cell RNA sequencing.

F - H. Histograms showing permutation analyses of 10000 iterations of randomly generated gene lists of equal size to gene list of DEgenes from bulk RNAseq, and ASD-related WGCNA from Parikshak, 2013 (4) (F), Willsey, 2013 (5) (G), Duda, 2018 (6) (H).Displayed are z-scores and two-sided p-values of the permutation tests.

I and J. Histograms showing permutation analyses of 10000 iterations of randomly generated gene lists of equal size to gene list of differentially expressed genes from the bulk RNAseq, compared to WGCNAs for AD (7) (I) and BD (8) (J). Displayed are z-scores and two-sided p-values of the permutation tests.

K - M. Histograms showing permutation analyses of 10000 iterations of randomly generated gene lists of equal size to a list of DE genes from single-cell RNAseq, and ASD-related WGCNA from Parikshak, 2013 (4) (F), Willsey, 2013 (5) (G), Duda, 2018 (6) (M). Enrichment for these DEgenes at the single-cell level is more pronounced than at the bulk RNAsequencing level shown in panel F-H. Displayed are z-scores and two-sided p-values of the permutation tests.

N - P. Enrichment of the 100 cell type differentiating genes from each *CNTNAP2*-expressing cell types for disease phenotypes as curated by DisGeNET(9). The results are sorted by the smallest FDR p-value.

Supplementary figure 4.

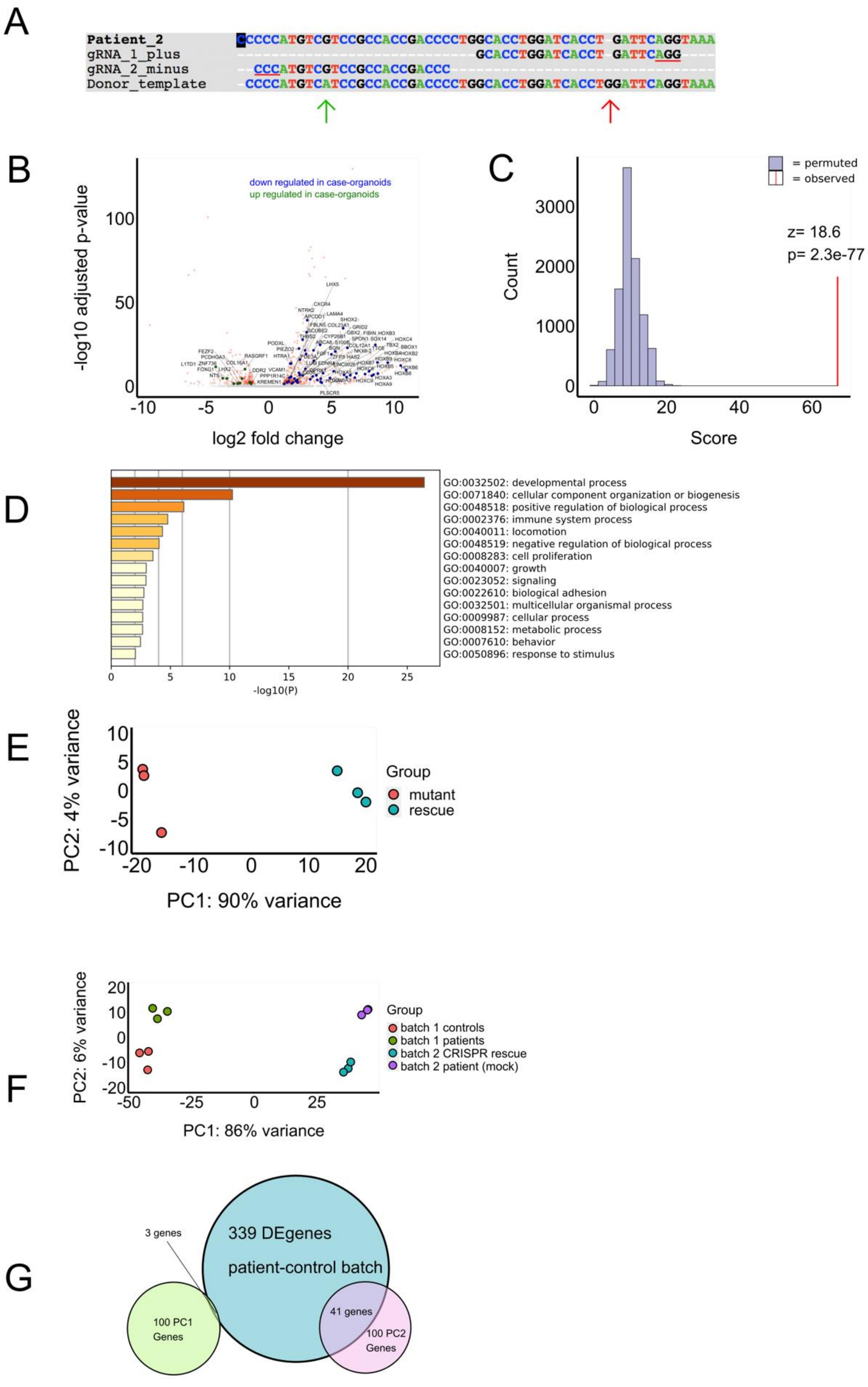

A
Patient_2
gRNA_1_plus
gRNA_2_minus
Donor_template
CCCCCATGTCGTCCGCCACCGACCCCTGGCACCTGGATCACCT GATTCAGGTAAA
GCACCTGGATCACCT GATTCAGG
CCCATGTCGTCCGCCACCGACCC
CCCCATGTCATCCGCCACCGACCCCTGGCACCTGGATCACCTGGATTCAGGTAAA
B
down regulated in case-organoids
up regulated in case-organoids
-log10 adjusted p-value
log2 fold change
C
= permuted
= observed
z= 18.6
p= 2.3e-77
Count
Score
D
GO:0032502: developmental process
GO:0071840: cellular component organization or biogenesis
GO:0048518: positive regulation of biological process
GO:0002376: immune system process
GO:0040011: locomotion
GO:0048519: negative regulation of biological process
GO:0008283: cell proliferation
GO:0040007: growth
GO:0023052: signaling
GO:0022610: biological adhesion
GO:0032501: multicellular organismal process
GO:0009987: cellular process
GO:0008152: metabolic process
GO:0007610: behavior
GO:0050896: response to stimulus
-log10(P)
E
Group
mutant
rescue
PC2: 4% variance
PC1: 90% variance
F
Group
batch 1 controls
batch 1 patients
batch 2 CRISPR rescue
batch 2 patient (mock)
PC2: 6% variance
PC1: 86% variance
G
3 genes
339 DEgenes
patient-control batch
41 genes
100 PC1 Genes
100 PC2 Genes

Supplementary figure 4 legend

A. CRISPR design showing the genomic location of the mutant DNA sequence of patient 2 and the targets of designed gRNAs. The Protospacer adjacent motif (PAM) sequence is underlined in red. The donor template has a G base inserted at cDNA position 3709 (red arrow) and a silent blocking mutation 6 base-pairs downstream from the gRNA2 PAM sequence (green arrow).

B. Volcano plot displaying the differentially expressed genes between CRISPR-rescue organoids and its parental patient-derived organoid line at 8 weeks *in vitro.* DE genes reaching statistical significance are colored in shaded red, and genes below statistical significance threshold are colored in shaded grey. 386 genes were upregulated while 190 genes were downregulated. Genes that were downregulated in case organoids but upregulated in the CRISPR rescue line (57 genes) are indicated as blue dots, genes that were upregulated in case organoids but are downregulated in the CRISPR-rescue line (10 genes) are indicated as green dots, indicating a partial rescue of the transcriptional profile in the CRISPR rescue line.

C. Permutation analysis (n=10,000) indicating that the partial rescue at the transcriptional level is many times more as would be expected from random events.

D. Gene Ontology (GO) analysis on the 67 genes that are highlighted in panel B of this figure. The results show the biological process ontologies sorted by the smallest FDR p-

value, and implicate multiple processes related to neurodevelopment including cellular proliferation.

E. Principal Component Analysis (PCA) shows a clear distinction between the expression profiles of CRISPR-rescue compared to it parental patient-derived organoids at 8 weeks *in vitro*.

F. PCA analysis showing that genotypes (Patient vs control and CRISPR-rescue vs patient) separate in a similar manner on PC2, which explains 6% of the variance. This analysis shows a large batch effect, with the two batches being clearly separated on the axis representing PC1 that explains 86% of the variance.

G. Circle diagram displaying that of the 339 genes differential expressed between patient- and control-organoids (Fig. 4A), 41 are present in the top-100 contributing genes to PC2 of panel F of this figure, where only 3 of the 339 DE genes are present in the top-100 contributing genes to PC1. This underscores that the separation on the horizontal axis is a batch effect that involves different genes than the 339 DE genes from the control-patient batch.

Supplementary References

1. Group WMGRS. Growth Standards: Growth velocity based on weight, length and head circumference: Methods and development. Geneva: World Health Organization 2009.
2. Nowakowski TJ, Bhaduri A, Pollen AA, Alvarado B, Mostajo-Radji MA, Di Lullo E, et al. Spatiotemporal gene expression trajectories reveal developmental hierarchies of the human cortex. Science. 2017;358(6368):1318-23.
3. Miller JA, Ding SL, Sunkin SM, Smith KA, Ng L, Szafer A, et al. Transcriptional landscape of the prenatal human brain. Nature. 2014;508(7495):199-206.

4. Parikshak NN, Luo R, Zhang A, Won H, Lowe JK, Chandran V, et al. Integrative functional genomic analyses implicate specific molecular pathways and circuits in autism. Cell. 2013;155(5):1008-21.
5. Willsey AJ, Sanders SJ, Li M, Dong S, Tebbenkamp AT, Muhle RA, et al. Coexpression networks implicate human midfetal deep cortical projection neurons in the pathogenesis of autism. Cell. 2013;155(5):997-1007.
6. Duda M, Zhang H, Li HD, Wall DP, Burmeister M, Guan Y. Brain-specific functional relationship networks inform autism spectrum disorder gene prediction. Transl Psychiatry. 2018;8(1):56.
7. Miller JA, Oldham MC, Geschwind DH. A systems level analysis of transcriptional changes in Alzheimer's disease and normal aging. J Neurosci. 2008;28(6):1410-20.
8. Chen H, Wang N, Zhao X, Ross CA, O'Shea KS, McInnis MG. Gene expression alterations in bipolar disorder postmortem brains. Bipolar Disord. 2013;15(2):177-87.
9. Pinero J, Ramirez-Anguita JM, Sauch-Pitarch J, Ronzano F, Centeno E, Sanz F, et al. The DisGeNET knowledge platform for disease genomics: 2019 update. Nucleic Acids Research. 2020;48(D1):D845-D55.
