# Supplementary material for: Cortical overgrowth in a preclinical forebrain organoid model of CNTNAP2-associated autism spectrum disorder
Source: Nat Commun. 2021 Sep 1;12:4087. doi: 10.1038/s41467-021-24358-4 (PMC8410758; doi:10.1038/s41467-021-24358-4)
Supplement: Supplementary file 3 — Description of Additional Supplementary Files [file 41467_2021_24358_MOESM3_ESM.pdf]

Description of Additional Supplementary Files

Supplementary data 1: Antibodies

Supplementary data 2: Primers and CRISPR design

Supplementary data 3: Patient MRI data

Supplementary data 4: CNTNAP2_5S.log1p_liger_singleR_Rank_leiden (unsupervised Leiden clusters)

Supplementary data 5: CNTNAP2_5S.log1p_liger_singleR_Rank_nowakowski (supervised clusters)

Supplementary data 6: CNTNAP2 DEgenes padj < 0.05

Supplementary data 7: GO_Upregulated DEgenes CNTNAP2

Supplementary data 8: GO_Downregulated CNTNAP2

Supplementary data 9: CNTNAP2 Overlap DEgenes CNTNAP2 and SFARI genes 1-4-syndromic

Supplementary data 10: Pseudo bulk CNTNAP2_5S.log1p_liger_singleR_nowakowski_3cellTypes_MAST

Supplementary data 11: Overlap DEgenes CNTNAP2 scRNAseq SFARI genes 1-4-syndromic

Supplementary data 12: Corpus callosum volume
