# Supplementary material for: Cortical overgrowth in a preclinical forebrain organoid model of CNTNAP2-associated autism spectrum disorder
Source: Nat Commun. 2021 Sep 1;12:4087. doi: 10.1038/s41467-021-24358-4 (PMC8410758; doi:10.1038/s41467-021-24358-4)
Supplement: Supplementary file 16 — Reporting summary [file 41467_2021_24358_MOESM16_ESM.pdf]

nature research

Corresponding author(s): Bin Xu and Sander Markx

Last updated by author(s): 2021-5-28

Reporting Summary

Nature Research wishes to improve the reproducibility of the work that we publish. This form provides structure for consistency and transparency in reporting. For further information on Nature Research policies, see our Editorial Policies and the Editorial Policy Checklist.

Statistics

For all statistical analyses, confirm that the following items are present in the figure legend, table legend, main text, or Methods section.

n/a	Confirmed	
☐	☒	The exact sample size (n) for each experimental group/condition, given as a discrete number and unit of measurement
☐	☒	A statement on whether measurements were taken from distinct samples or whether the same sample was measured repeatedly
☐	☒	The statistical test(s) used AND whether they are one- or two-sided<br>*Only common tests should be described solely by name; describe more complex techniques in the Methods section.*
☒	☐	A description of all covariates tested
☐	☒	A description of any assumptions or corrections, such as tests of normality and adjustment for multiple comparisons
☐	☒	A full description of the statistical parameters including central tendency (e.g. means) or other basic estimates (e.g. regression coefficient) AND variation (e.g. standard deviation) or associated estimates of uncertainty (e.g. confidence intervals)
☐	☒	For null hypothesis testing, the test statistic (e.g. F, t, r) with confidence intervals, effect sizes, degrees of freedom and P value noted<br>*Give P values as exact values whenever suitable.*
☒	☐	For Bayesian analysis, information on the choice of priors and Markov chain Monte Carlo settings
☒	☐	For hierarchical and complex designs, identification of the appropriate level for tests and full reporting of outcomes
☐	☒	Estimates of effect sizes (e.g. Cohen's d, Pearson's r), indicating how they were calculated

Our web collection on statistics for biologists contains articles on many of the points above.

Software and code

Policy information about availability of computer code

Data collection	-CT-Finder software (online)
Data analysis	- DESeq Version 1.28.1<br>- R Version 4.0.0<br>- ImageJ Version 2.0.0-rc-69/1.52p (https://imagej.nih.gov/ij/).<br>- Cell profiler Version 4.1.3<br>- SCANPY Version 1.7.1<br>- SingleR Version 1.0.6<br>- MAST algorithm Version 1.12.0<br>- LIGER Version 2.0.1<br>- Cell Ranger pipeline Version 4.0.0 (10x Genomics) \<br>- Metascape (online)

For manuscripts utilizing custom algorithms or software that are central to the research but not yet described in published literature, software must be made available to editors and reviewers. We strongly encourage code deposition in a community repository (e.g. GitHub). See the Nature Research guidelines for submitting code & software for further information.

Data

Policy information about availability of data

All manuscripts must include a data availability statement. This statement should provide the following information, where applicable:

- Accession codes, unique identifiers, or web links for publicly available datasets
- A list of figures that have associated raw data
- A description of any restrictions on data availability

Bulk and singel cell RNA sequencing transcriptome data are available under accession code: GSE174569
Publicly available data used in this paper include:
- Brainspan transcriptome database (Miller JA, Ding SL, Sunkin SM, Smith KA, Ng L, Szafer A, et al. Transcriptional landscape of the prenatal human brain. Nature. 2014;508(7495):199-206.) www.brainspan.org
- human embryonic PFC single cell transcriptome (Nowakowski TJ, Bhaduri A, Pollen AA, Alvarado B, Mostajo-Radji MA, Di Lullo E, et al. Spatiotemporal gene expression trajectories reveal developmental hierarchies of the human cortex. Science. 2017;358(6368):1318-23.) www.sciencemag.org/content/358/6368/1318/suppl/DC1
- predefined cell type markers (Bhaduri, A., Andrews, M. G., Mancia Leon, W., Jung, D., Shin, D., Allen, D., Jung, D., Schmunk, G., Haeussler, M., Salma, J., Pollen, A. A., Nowakowski, T. J., & Kriegstein, A. R. (2020). Cell stress in cortical organoids impairs molecular subtype specification. Nature, 578(7793), 142–148. https://doi.org/10.1038/s41586-020-1962-0). suppl table 2.

Figures associated with available raw data:
Figure 1E, 1F, 4A, 4B, 4C, 4D, 4E, 4F, 5B, 5G, S1J, S3A-P, S4B-G.

Field-specific reporting

Please select the one below that is the best fit for your research. If you are not sure, read the appropriate sections before making your selection.

- [x] Life sciences
- [ ] Behavioural & social sciences
- [ ] Ecological, evolutionary & environmental sciences

For a reference copy of the document with all sections, see nature.com/documents/nr-reporting-summary-flat.pdf

Life sciences study design

All studies must disclose on these points even when the disclosure is negative.

Sample size	We used a sample size of 3 patient-derived cell lines, and 3 control-derived cell lines to generate cortical organoids. We then used biological replicates ranging between n=4 and n=10 per line. We chose the number of biological replicates per line based on conventional power analyses.<br><br>For the experiments not involving cell lines we did not use power analyses to determine sample size. For the MRI-analysis we used the number of MRI scans we could obtain (n=6). For the head circumference measurements we used the all data we could obtain through the Clinic for Special Children in Strasburg, PA (n=37) and compared this to WHO reference data.
Data exclusions	For the scRNAseq experiment performed in this study, one control sample ('C3') run failed during the experiment due to technical issues
Replication	We used biological replicates (n=4-10) for most experiments in this study, which have been shown as individual points in the box plots, with different colors for different cell lines.<br><br>The experiment investigating organoid size based on 2D light microscopy images was replicated once in its entirety and yielded the same results. Other experiments included in the study were performed one time.
Randomization	No randomization was performed in this study. Covariates were controlled by ensuring identical treatment of case- and control groups: Organoids from different genotype groups were kept on the same shelve in the incubator, treated with the same media and processed in the same way.
Blinding	Image analysis was done in a blinded fashion for the Brdu/Ki76 experiment (Fig 2C-D, S2D) and the neuronal soma size experiment (Fig S2B)<br><br>For the remaining experiments (isotropic fractionation, organoid size, bulk and single -cell RNAseq) it was not possible to maintain blindness for the investigator handling the organoids, as the organoid volume phenotype between case and control organoids is robust to such an extent that the genotype can be identified by looking at the size of the organoid.

Reporting for specific materials, systems and methods

We require information from authors about some types of materials, experimental systems and methods used in many studies. Here, indicate whether each material, system or method listed is relevant to your study. If you are not sure if a list item applies to your research, read the appropriate section before selecting a response.

Materials & experimental systems

n/a	Involved in the study
☐	☒ Antibodies
☐	☒ Eukaryotic cell lines
☒	☐ Palaeontology and archaeology
☒	☐ Animals and other organisms
☐	☒ Human research participants
☒	☐ Clinical data
☒	☐ Dual use research of concern

Methods

n/a	Involved in the study
☒	☐ ChIP-seq
☒	☐ Flow cytometry
☐	☒ MRI-based neuroimaging

Antibodies

Antibodies used

Also See table s1 for full list of antibodies used in the study

Anti-MAP2 Abcam Ab5392 Chicken
Anti-PAX6 Biolegend 901302 Rabbit
Anti-CNTNAP2 A Genscript A01426-100 Rabbit
Anti-CNTNAP2 B K67/25 Neuromab #75-075 Mouse
Anti-Ki67 Abcam Ab6326 Rabbit
Anti-BrdU Abcam Ab6326 Rat
Anti-TBR1 Abcam Ab31940 Rabbit
Anti-CTIP2 Abcam Ab18465 Rat
Anti-FOXG1 Abcam Ab196868 Rabbit
Anti-BrdU Abcam Ab6326 Rat
Anti-!-Actin Sigma-Aldrich A5441 Mouse
Anti-GAPDH Sigma-Aldrich G9545 Rabbit

Validation

-Abcam states antibodies are KO-validated on their website.
-Anti-MAP2 Abcam Ab5392 Chicken:ab5392 has been referenced in 444 publications.
-Anti-Ki67 Abcam Ab6326 Rabbit: has been knock-out validated. ab15580 has been referenced in 2170 publications.
-Anti-BrdU Abcam Ab6326 Rat: validated by staining on Brdu-negative organoid: no signal
-Anti-TBR1 Abcam Ab31940 Rabbit:ab31940 has been referenced in 278 publications.
-Anti-CTIP2 Abcam Ab18465 Rat:ab18465 has been referenced in 445 publications.
-Anti-FOXG1 Abcam Ab196868 Rabbit. ab18259 has been referenced in 74 publications.
-Labome states CNTNAP2 Mouse antibody is KO-validated. Western blots in our study confirmed KO at the expected molecular weight,
including reverse-KO validation using the CRISPR rescue line
-Genscript CNTNAP2 Rabbit antibody does not have a KO-validation statement on their website. Western blots in our study confirmed KO at the expected molecular weight
- The B-Actin antibody from Sigma was used in 7111 peer reviewed studies, the website does not state a KO-validation process
- The GAPDH antibody from Sigma was used in 994 peer reviewed studies, the website does not state a KO-validation process
- The PAX6 antibody was referenced in multiple application references: https://www.biolegend.com/en-us/global-elements/pdfpopup/
purified-anti-pax-6-antibody-11511?filename=Purified%20anti-Pax-6%20Antibody.pdf&pdfgen=true

Eukaryotic cell lines

Policy information about cell lines

Cell line source(s)	human induced pluripotent stem cells were generated from individuals we recruited from the Old Order Amish population
Authentication	None of the cell lines used in this study were authenticated
Mycoplasma contamination	The cell lines used in this study have been tested for mycoplasma contamination. There was no mycoplasma contamination.
Commonly misidentified lines (See ICLAC register)	No commonly misidentified cell lines were used in the study

Human research participants

Policy information about studies involving human research participants

Population characteristics	All cell lines (both the 3 female cases and 3 female controls) were derived from the Old-Order Amish founder population in Pennsylvania through collaboration with the Clinic for Special Children where these subjects are being followed for clinical care and participation in research studies.
Recruitment	Participants were recruited from the Old Order Amish population based on a diagnosis with cortical dysplasia focal epilepsy syndrome and confirmed genotype for CNTNAP2. Control-participants were recruited from the Old Order Amish population based on age, sex and willingness to participate. Cases and controls were matched based on these characteristics in order to reduce the effect of these variables on the study outcomes
Ethics oversight	The study was approved by the Lancaster General Hospital (LGH) Institutional Review Board (IRB) (LGH IRB protocol number 2008-095).<br>The study design using these iPSC lines was approved by Columbia University's Human Embryo and Embryonic Stem Cell Research Committee

Note that full information on the approval of the study protocol must also be provided in the manuscript.

Magnetic resonance imaging

Experimental design

Design type	Retrospective analysis of structural volumetric changes
Design specifications	N/A as this was not an fMRI study
Behavioral performance measures	N/A as this was not an fMRI study

Acquisition

Imaging type(s)	Structural
Field strength	1.5, 3.0 T
Sequence & imaging parameters	There was no fixed sequence or imaging parameters for the clinical scans, which include both T1 and T2-weighted structural whole brain imaging. The template scans were obtained having been generated from T1-weighted structured MRI scans, and as such, the template images do not have a sequence.
Area of acquisition	Whole Brain

Diffusion MRI ☐ Used ☒ Not used

Preprocessing

Preprocessing software	FSL 5.0 - FLIRT, SPM12 on MATLAB R2017b.
Normalization	Each subject MRI scan was co-registered to the appropriate age-matched template space.
Normalization template	Average templates were obtained, not generated, from original sources.
Noise and artifact removal	Noise and artifact removal was not used as this was not an fMRI study
Volume censoring	Volume cencoring was not performed for the structural MRI study

Statistical modeling & inference

Model type and settings	No quantitative fMRI analysis was performed.
Effect(s) tested	This is not applicable as no quantitative fMRI analysis was performed.

Specify type of analysis: ☐ Whole brain ☐ ROI-based ☐ Both

Statistic type for inference (See Eklund et al. 2016)	This is not applicable as no quantitative fMRI analysis was performed.
Correction	This is not applicable as no quantitative fMRI analysis was performed.

Models & analysis

n/a	Involved in the study
☒	☐ Functional and/or effective connectivity
☒	☐ Graph analysis
☒	☐ Multivariate modeling or predictive analysis
